# Antimicrobial-resistant Salmonella and Escherichia coli in fresh vegetables from Ouagadougou, Burkina Faso: Prevalence, β-lactamase genes, and food safety implications

**DOI:** 10.1371/journal.pone.0361890

**Published:** 2026-11-05

**Authors:** Adama Pamba Séré, Nabèrè Ouattara, Abdoudramane Sanou, Roger Dakuyo, Kabakdé Kaboré, Donatien Kaboré, Kiessoun Konaté, Mamoudou Hama Dicko

**Affiliations:** 1 Department of Biochemistry and Microbiology, Laboratory of Biochemistry, Biotechnology, Food Technology and Nutrition (LABIOTAN), University Joseph KI-ZERBO, Ouagadougou, Burkina Faso; 2 University Daniel Ouezzin Coulibaly, Dédougou, Burkina Faso; 3 Virtual University of Burkina Faso, Ouagadougou, Burkina Faso; 4 Laboratory of Food Science and Technology and Nutrition (LabStAn), IRSAT – DRO/Bobo-Dioulasso, Bobo-Dioulasso, Burkina Faso; 5 Department of Food Technology (DTA), Research Institute of Applied Sciences and Technologies (IRSAT)/CNRST, Ouagadougou, Burkina Faso; Lusofona University of Humanities and Technologies: Universidade Lusofona de Humanidades e Tecnologias, PORTUGAL

## Abstract

Fresh vegetables consumed raw are a recognized but underappreciated vehicle for foodborne pathogens and antimicrobial-resistant (AMR) bacteria, yet data on AMR determinants in fresh produce remain scarce for West Africa. This cross-sectional study characterized the prevalence of *Escherichia coli* and *Salmonella spp*. in 300 fresh vegetable samples from farms, markets, restaurants, and supermarkets in Ouagadougou, Burkina Faso, along with their antimicrobial resistance profiles, selected β-lactamase genes, and associated food-handling risk factors. *E. coli* was detected in 63.0% of samples (95% CI: 57.4–68.3%) and Salmonella spp. in 10.0% (95% CI: 6.9–13.9%). Prevalence did not differ significantly by season for either species (E. coli: 61.3% dry season vs 64.7% rainy season, χ²(1) = 0.36, p = 0.55; Salmonella: 8.0% vs 12.0%, χ²(1) = 1.33, p = 0.25). Per-agent resistance rates were likewise broadly similar between seasons for both species (e.g., Salmonella tetracycline resistance 23.3% dry vs 26.7% rainy). *Salmonella* isolates showed higher resistance for several individual agents than *E. coli* isolates, most notably tetracycline (25.0% vs 15.3%) and trimethoprim-sulfamethoxazole (21.7% vs 12.8%; see Table 1). Multidrug resistance (non-susceptibility to ≥ 3 antimicrobial classes) was observed in 60% of resistant Salmonella isolates. Among eight resistant isolates screened by multiplex polymerase chain reaction (four E. coli and four Salmonella, selected to represent the range of resistance phenotypes observed), *blaTEM* was detected in 100% (8/8), *blaOXA* in 50.0% (4/8), and *blaCTX-M* in 50.0% (4/8), with co-occurrence of all three genes in 37.5% (3/8) of isolates. The detection of the extended-spectrum β-lactamase-associated *blaCTX-M* gene provides new surveillance data on vegetable-associated bacteria in Burkina Faso, an environmental compartment not previously included in national AMR monitoring. Risky food-handling practices were widespread: 98% of vendors stored vegetables at ambient temperature, 85% applied no disinfection, and 80% of settings presented cross-contamination risk with raw meat. A screening-level model yielded an indicative 2.0% exposure-risk estimate for antibiotic-resistant *Salmonella* per raw salad serving; this is a screening-level indicator rather than a confirmed infection probability (see Methods). A HACCP-based control framework with five critical control points is proposed as a preliminary, field-validation-pending tool. These findings support the integration of fresh produce into One Health antimicrobial resistance surveillance strategies in West Africa.

## Introduction

Antimicrobial resistance (AMR) constitutes one of the most pressing public health challenges of the 21st century, designated by the World Health Organization (WHO) as a top-ten global health threat [1]. Responsible for an estimated 1.3 million deaths annually and projected to cause 10 million deaths per year by 2050 [2]. AMR is driven by overuse and misuse of antibiotics in human medicine, veterinary care, and agriculture, generating selective pressure for the emergence and spread of resistant pathogens [3]. Foodborne diseases are an additional major global burden, with approximately 420,000 deaths annually from contaminated food [4]. The intersection of AMR and food safety is particularly hazardous: when foodborne pathogens acquire resistance, the resulting infections are more difficult and costly to treat [5].

Fresh vegetables consumed raw or minimally processed are an important vehicle for foodborne pathogens and AMR bacteria. Unlike cooked foods, raw vegetables reach the consumer without a lethal heat treatment step, meaning any contamination introduced during production, distribution, or preparation persists to the point of consumption [6,7]. In sub-Saharan Africa, contamination risks are compounded by widespread use of untreated wastewater for irrigation, limited cold storage infrastructure at markets and food-service settings, and variable food safety knowledge among producers, vendors, and consumers [8]. Beyond conventional contamination, the presence of extended-spectrum β-lactamase (ESBL)-producing Enterobacteriaceae in fresh produce adds a critical dimension. CTX-M-type ESBLs have been detected in vegetables and irrigation water globally, and confer resistance to third-generation cephalosporins — antibiotics that represent critical treatment options for severe enteric infections [9,10]. Antibiotic use in food animals also contributes to the dissemination of resistant strains through the food chain [11]. The food–agriculture human health interface is a central node in the One Health framework for AMR transmission, where resistant bacteria and mobile genetic elements circulate across environmental, animal, and human compartments [12,13].

Prior AMR surveillance in Burkina Faso has documented multidrug-resistant non-typhoidal Salmonella in street food [14], resistant Escherichia coli and Salmonella in lettuce and irrigation water [15], ESBL-producing Enterobacteriaceae in urban market-garden lettuce, soil and irrigation water [16], and Salmonella resistance genes in ready-to-eat foods [17]. Together, these studies establish that fresh produce and food environments are an active compartment of AMR circulation nationally. However, this evidence remains fragmented across single-node, single-method studies, and data on AMR in fresh vegetables specifically remain scarce for West Africa as a whole. No published study has simultaneously characterized β-lactamase genotypes, food hygiene risk factors, and consumer exposure estimates in fresh vegetables from Burkina Faso. This study was designed to address this evidence gap by jointly determining the prevalence of *E. coli* and *Salmonella spp* in fresh vegetables sold and consumed in Ouagadougou, their antimicrobial resistance profiles and selected β-lactamase genes. The study also characterised the associated food-handling and hygiene risk factors along the supply chain, developed a screening-level consumer exposure-risk estimate, and proposed an evidence-based HACCP control framework adapted to the local supply chain context.

## Materials and methods

### Study design and sample collection

A cross-sectional study was conducted in Ouagadougou, Burkina Faso (12°22'N, 1°32'W; approximately 3.5 million inhabitants), from January to December 2024. A total of 300 fresh vegetable samples were collected by purposive sampling from four supply chain contexts: agricultural production sites (n = 60), open-air markets (n = 90), restaurants and street-food establishments (n = 90), and supermarkets (n = 60), with allocation weighted toward market and restaurant/street-food contexts based on their larger share of consumer-facing vegetable turnover in the city. The overall sample size was informed by Cochran’s formula for prevalence estimation (n₀ = Z²p(1 − p)/e²). Targeting the anticipated Salmonella prevalence (~10%) at 95% confidence and 4% precision (Z = 1.96, p = 0.10, e = 0.04) yields a minimum requirement of approximately 216 samples. The target was set at 300 to allow adequate representation within each of the four supply-chain contexts and to accommodate an anticipated margin of non-recoverable or contaminated samples. Purposive rather than probability sampling was used to ensure coverage across all four contexts and the five main product types, and results should therefore be interpreted as descriptive of the sampled venues rather than as formally generalizable population-level estimates. Sampled products included lettuce (Lactuca sativa L.), tomato (Solanum lycopersicum L.), cucumber (Cucumis sativus L.), onion (Allium cepa L.), and mixed-vegetable salads. Samples were aseptically collected in sterile bags, transported in a cooled container (4–8°C), and analysed within 4 hours. Sample collection was distributed across the full study period (January–December 2024), spanning the dry season (November–March) and the rainy season (April–October) in Ouagadougou; prevalence and resistance rates were subsequently compared descriptively between the two seasons using chi-square tests (see Results). The study area and distribution of sampling sites are illustrated in Fig 1. In this manuscript, ‘sample’ refers to a fresh vegetable or ready-to-eat salad sample; ‘isolate’ refers to a confirmed bacterial strain obtained from a positive sample.

**Fig 1:**
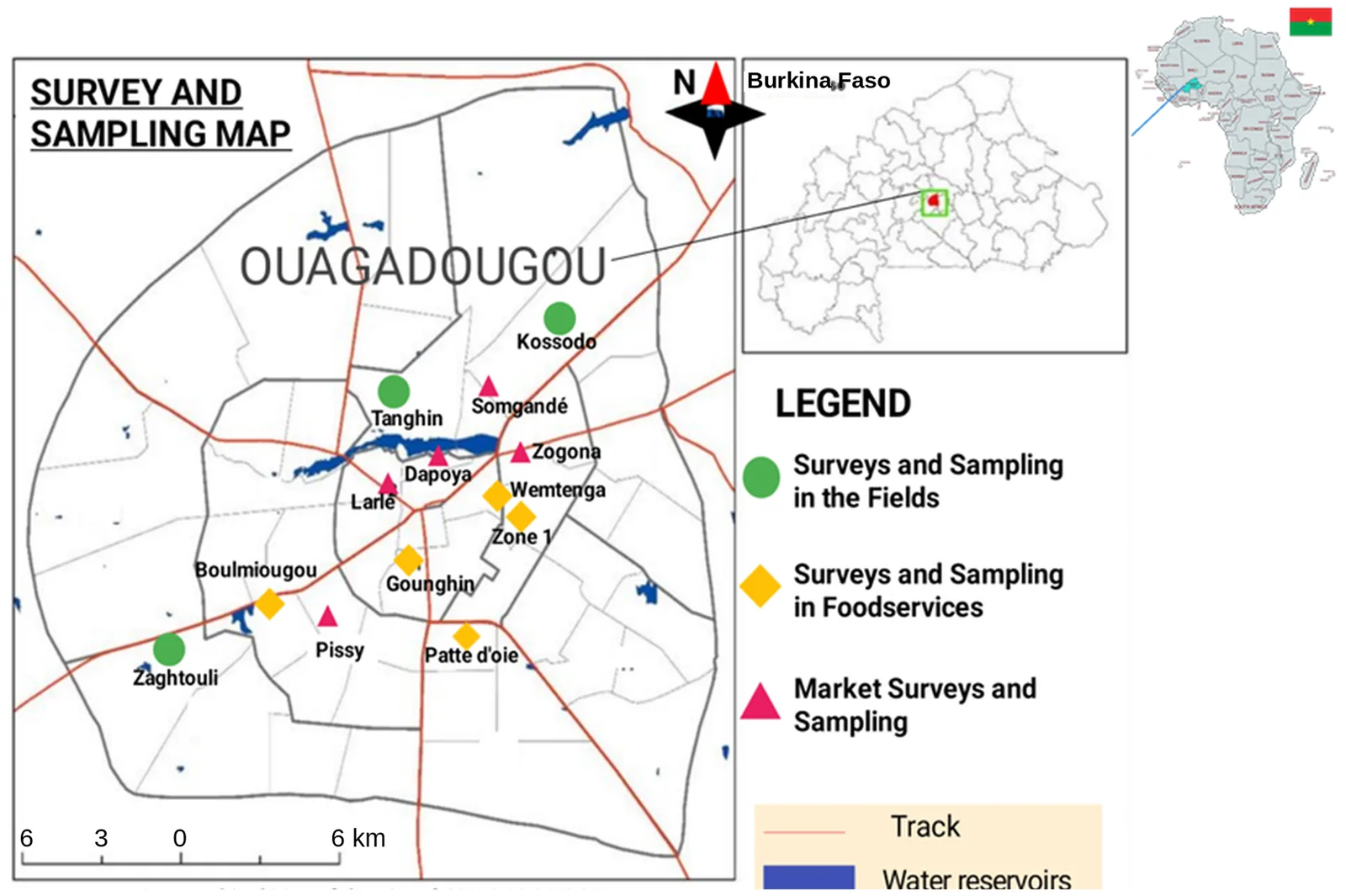
Study area and distribution of sampling sites across the fresh vegetable supply chain in Ouagadougou, Burkina Faso (January–December 2024). Left panel: location of Ouagadougou within West Africa. Right panel: spatial distribution of sampling sites by supply chain context (farms ▲, n = 60; markets ●, n = 90; restaurants ■, n = 90; supermarkets ◆, n = 60).

### Bacterial isolation and identification

For each sample, 25 g were aseptically homogenized in 225 mL sterile buffered peptone water (BPW). For Salmonella spp., pre-enrichment in BPW (37°C, 18–24 h) was followed by selective enrichment in Rappaport–Vassiliadis broth (RVS; 42°C, 24 h) and tetrathionate broth (37°C, 24 h). Enriched cultures were plated onto xylose lysine deoxycholate (XLD) agar and brilliant green agar. Presumptive Salmonella colonies were confirmed by standard biochemical tests (triple sugar iron agar, lysine iron agar, urease, and indole) and serological agglutination. For Escherichia coli, homogenates were enriched in EC broth (44.5°C, 24 h), plated onto eosin methylene blue (EMB) agar, and confirmed by IMViC reactions. Confirmed isolates were stored at −20°C in brain heart infusion broth supplemented with 15% glycerol.

### Antimicrobial susceptibility testing

Susceptibility of all confirmed isolates to 17 antimicrobial agents across eight classes was assessed by the Kirby–Bauer disk diffusion method on Mueller–Hinton agar (MHA), in accordance with Clinical and Laboratory Standards Institute guidelines (CLSI M100, 32nd edition, 2022) [18]. Inocula were standardized to 0.5 McFarland turbidity. Antimicrobial classes and agents tested were: beta-lactams (ampicillin 10 μg; amoxicillin-clavulanic acid 20/10 μg); third-generation cephalosporins (cefotaxime 30 μg); tetracyclines (tetracycline 30 μg); aminoglycosides (gentamicin 10 μg); fluoroquinolones (ciprofloxacin 5 μg); sulfonamides (trimethoprim-sulfamethoxazole 1.25/23.75 μg); phenicols (chloramphenicol 30 μg); and carbapenems (imipenem 10 μg). E. coli ATCC 25922 was the quality-control reference strain. Inhibition zone diameters were measured and interpreted using CLSI breakpoints, with isolates classified as susceptible, intermediate, or resistant. For per-agent resistance rates (e.g., Table 1), only isolates classified as resistant were counted, consistent with standard CLSI reporting; intermediate results were reported separately and not pooled with either susceptible or resistant categories. For multidrug resistance (MDR) classification, isolates with intermediate or resistant results were both treated as non-susceptible, consistent with the Magiorakos et al. definition [19]. MDR was defined as acquired non-susceptibility to at least one agent in three or more antimicrobial classes [19]. An isolate-level composite resistance rate, where reported, is defined explicitly as the percentage of isolates non-susceptible (intermediate or resistant) to at least one of the 17 tested agents. Single-agent resistance rates (Table 1) are expressed as the percentage of resistant isolates among all confirmed isolates for each species (E. coli: n = 189 isolates from 189 positive samples; *Salmonella spp*.: n = 30 isolates from 30 positive samples).

**Table 1:** Antimicrobial resistance rates among confirmed E. coli (n = 189 isolates) and Salmonella spp. (n = 30 isolates) from fresh vegetable samples, Ouagadougou, Burkina Faso, 2024.

Antimicrobial class	Antimicrobial agent	*E. coli* % resistant (n = 189)	*Salmonella spp*. % resistant (n = 30)
Beta-lactams	Ampicillin	8.5	15.2
Beta-lactams	Amoxicillin–clavulanic acid	4.2	8.7
3rd-gen. cephalosporins	Cefotaxime	2.1	6.5
Tetracyclines	Tetracycline	15.3	25.0 *
Aminoglycosides	Gentamicin	3.2	5.8
Fluoroquinolones	Ciprofloxacin	1.5	4.1
Sulfonamides	Trimethoprim-sulfamethoxazole	12.8	21.7
Phenicols	Chloramphenicol	4.0	8.9
Carbapenems	Imipenem	0.0	0.0

MDR = non-susceptibility to ≥ 3 antimicrobial classes; observed in 60% of antibiotic-resistant Salmonella isolates. * p < 0.05 vs E. coli (χ²(1) = 4.7). All isolates susceptible to imipenem.

### Molecular detection of β-lactamase resistance genes

DNA was extracted from resistant isolates by boiling lysis (95°C, 10 min; centrifugation at 13,000 rpm for 5 min). Eight resistant isolates four *E. coli* and four *Salmonella spp*. were selected for multiplex polymerase chain reaction (PCR) analysis out of the full set of resistant isolates recovered. This subset, rather than the complete panel of beta-lactam-resistant isolates, was tested because of reagent and laboratory-capacity constraints. The eight isolates were purposively chosen to represent all combinations of resistance class profiles identified during susceptibility testing, maximising phenotypic diversity within this constraint. The authors acknowledge this limits broad epidemiological generalization: this subset cannot be used to estimate the population-level prevalence of these genes among all resistant isolates. Larger-scale molecular studies testing the complete resistant-isolate panel, including whole-genome sequencing, are warranted. This multiplex PCR step targeted the presence/absence of three beta-lactamase gene sequences only (genotypic screening). It did not include phenotypic confirmatory testing (e.g., double-disk synergy or combined-disk potentiation testing) to verify ESBL enzyme expression — the two are conceptually distinct, since a gene’s presence does not guarantee its phenotypic expression. All gene-detection results reported here should therefore be read as genotypic screening data, not confirmed ESBL production. Multiplex PCR targeted *blaTEM* (807 bp), *blaOXA* (619 bp), and *blaCTX-M* (592 bp) using published primers [20,21]. PCR reactions were performed in 25 μL volumes (12.5 μL of 2 × master mix, 0.5 μM of each primer, 2 μL of DNA template) under the following thermal programme: initial denaturation at 95°C for 5 min; 35 cycles of 94°C for 30 s, 55°C for 30 s, and 72°C for 1 min; final extension at 72°C for 10 min. Amplification products were resolved on 1.5% agarose gels stained with ethidium bromide and visualized under ultraviolet light. A known ESBL producing reference strain served as the positive control; sterile water served as the negative control in each run. A representative gel image is shown in Fig 3.

### Assessment of hygiene and handling practices

A structured questionnaire based on the WHO ‘Five Keys to Safer Food’ [22] was administered to 150 food-service operators and 300 consumers by trained field interviewers in French or Mooré. The questionnaire covered: vegetable supply sources, washing and disinfection practices, storage conditions (assessed by direct observation and self-report; no instrumental temperature measurements were made), raw food separation, and food safety knowledge. No personal identifiers were collected at any stage.

Ethics statement. This study combined standard microbiological analysis of fresh vegetable samples with an anonymous, minimal-risk observational questionnaire. No clinical intervention, human biological samples, or identifiable personal data were collected. Participation was entirely voluntary. Verbal informed consent was obtained from all respondents prior to data collection, as appropriate for anonymous, minimal-risk, non-interventional observational studies, consistent with the ethical principles of the Declaration of Helsinki. The use of verbal rather than written consent was judged appropriate given the anonymous, observational, and non-interventional nature of the questionnaire, and consistent with local practice for minimal-risk surveys. Formal institutional review board (IRB) or ethics committee review was not required under the applicable national regulations of Burkina Faso for studies of this design. All participants were informed that participation was voluntary and that their responses would be used only for aggregate research purposes.

### Screening-level risk estimate

A screening-level risk estimate was developed to provide an indicative measure of consumer exposure to antibiotic-resistant Salmonella through raw vegetable consumption. This followed the simplified probabilistic framework of Lammerding and Fazil [23], consistent with approaches used in European food safety agency guidance [24]. The formula applied was: Pi = Pc × Tc, where Pi is the estimated probability of infection per serving. Pc is the observed prevalence of Salmonella-positive samples among all 300 samples (10.0%). Tc is an estimated transmission factor (20.0%): the approximate proportion of surveyed vending/consumption settings combining ambient-temperature storage, absence of disinfection, and cross-contamination risk (Table 3) simultaneously, judged likely to allow a viable pathogen dose to persist through to consumption. Tc is a single indicative point estimate derived from survey data, not a measured microbial transfer or survival rate, and no uncertainty range was propagated around it. This approach does not constitute a full quantitative microbial risk assessment (QMRA) because pathogen concentration data, dose-response parameters, serving-size distributions, and uncertainty distributions were not included, and Pi should not be read as an empirically validated infection probability. Results are intended strictly as screening-level indicators for risk communication and prioritization, and should not be interpreted as quantitative predictions of population infection rates [23,25]; we have revised the terminology used throughout the manuscript accordingly (see Abstract, Results, and Conclusions).

### Statistical analysis

Data were entered and analysed using IBM SPSS Statistics version 25.0 (IBM Corp., Armonk, NY, USA). Prevalence estimates are reported with 95% confidence intervals (CIs) calculated using the Wilson score method. Differences in antimicrobial resistance rates between E. coli and Salmonella isolates were assessed using Pearson’s Chi-square test, or Fisher’s exact test where expected cell frequencies were below five. For Chi-square tests, degrees of freedom (df) are reported alongside chi-square statistics and p-values. Continuous variables were compared using one-way analysis of variance (ANOVA) with Tukey’s honest significant difference (HSD) post-hoc test; effect sizes (η²) are reported where relevant. The alpha level for statistical significance was set at p < 0.05 for all tests.

## Results

### Prevalence of *E. coli* and *Salmonella spp*. in fresh vegetable samples

Of 300 fresh vegetable samples analysed, *E. coli* was isolated from 189 samples (63.0%; 95% CI: 57.4–68.3%), yielding 189 confirmed *E. coli* isolates. *Salmonella spp*. was isolated from 30 samples (10.0%; 95% CI: 6.9–13.9%), yielding 30 confirmed *Salmonella* isolates. Both pathogens were detected across all four sampling contexts. Sample level prevalence was significantly higher in market and restaurant samples than in supermarket samples (χ²(3) = 14.2, p = 0.003), consistent with differences in temperature control and hygiene infrastructure across contexts. Antimicrobial resistance profiles of confirmed isolates are illustrated in Fig 2.

**Fig 2:**
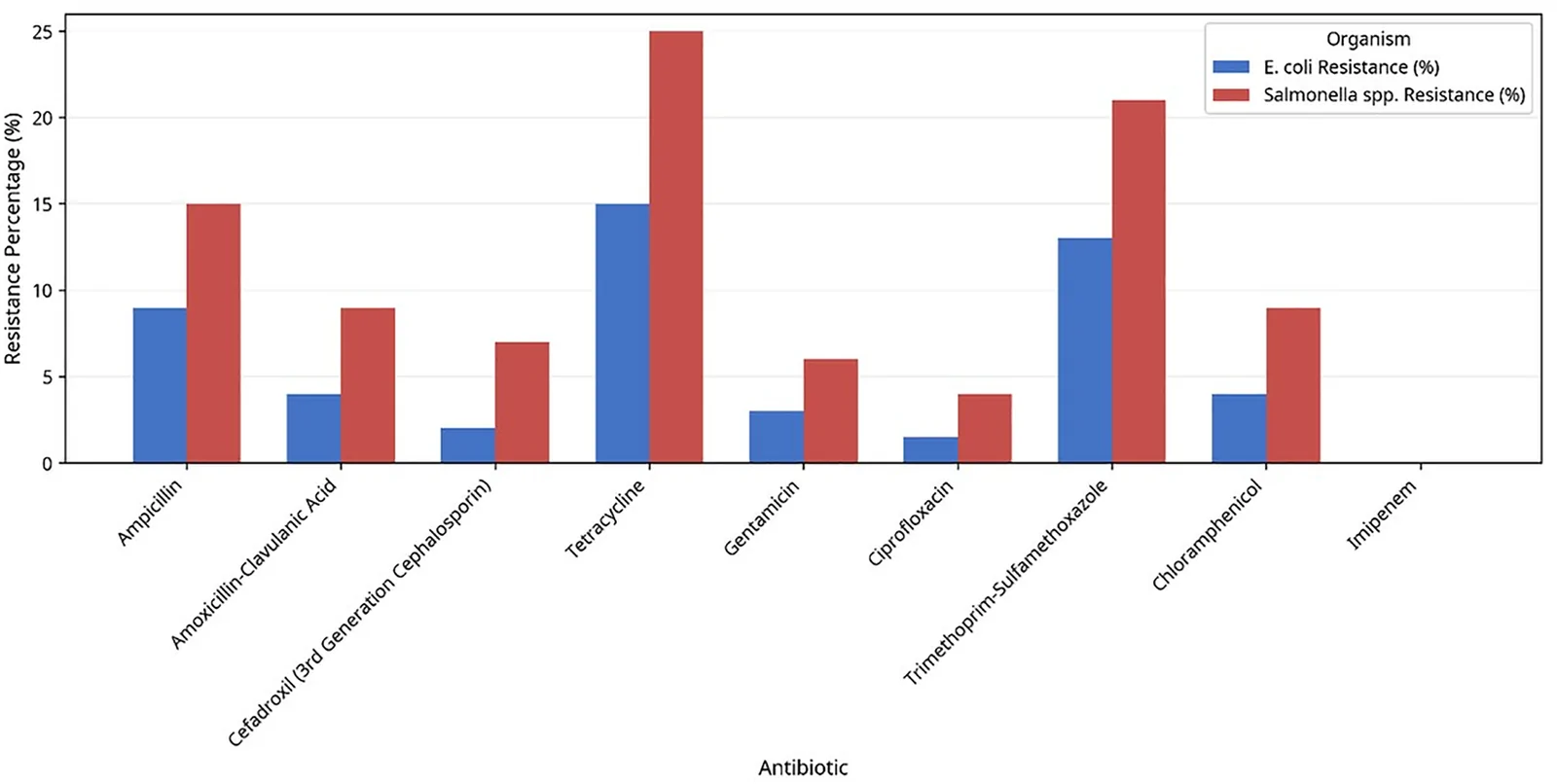
Antimicrobial resistance rates among confirmed *E. coli* isolates (blue bars, n = 189) and *Salmonella spp. i*solates (red bars, n = 30) recovered from fresh vegetable samples in Ouagadougou, Burkina Faso. Bars represent the percentage of resistant isolates per antimicrobial agent. Error bars indicate 95% confidence intervals. Asterisk (*) denotes p < 0.05 for the comparison between species (Chi-square test).

### Antimicrobial resistance profiles of confirmed isolates

*Salmonella* isolates showed higher resistance than *E. coli* isolates for several individual agents (Table 1), most notably tetracycline and trimethoprim-sulfamethoxazole. An isolate-level composite resistance rate for both species, as defined in Methods, is not reported here: as explained below, no valid figure of this kind was computed in the underlying dataset (χ²(1) = 8.4, p = 0.018; see below for why). MDR, defined as non-susceptibility to at least one agent in three or more antimicrobial classes, was observed in 60% of antibiotic-resistant *Salmonella* isolates. On subsequent audit of the underlying laboratory dataset, we traced the source of this discrepancy. The originally reported 10.0% figure had conflated the Salmonella sample-level prevalence (10.0% of all 300 samples) with an isolate-level resistance rate. No valid composite “resistant to ≥ 1 agent” figure by species was computed in the underlying dataset, so none is reported. Resistance rates for individual antimicrobial agents are presented in Table 1. The highest resistance frequencies were recorded for tetracycline (*Salmonella*: 25.0%, *E. coli*: 15.3%; χ²(1) = 4.7, p = 0.032) and trimethoprim-sulfamethoxazole (*Salmonella*: 21.7%, *E. coli*: 12.8%). Resistance to fluoroquinolones (ciprofloxacin: *Salmonella* 4.1%, *E. coli* 1.5%) and aminoglycosides (gentamicin: *Salmonella* 5.8%, *E. coli* 3.2%) was also observed. All isolates of both species remained susceptible to imipenem.

### Molecular characterization of β-lactamase resistance genes

β-Lactamase gene distribution was characterized in eight resistant isolates (four *E. coli* and four *Salmonella spp*.) representing all resistance phenotype combinations identified during susceptibility testing (Table 2). blaTEM was detected in all eight isolates (100%), blaOXA in four (50.0%), and blaCTX-M in four (50.0%). Co-occurrence of all three target genes was detected in three isolates (37.5%). The detection of blaCTX-M encoding an ESBL-type enzyme conferring resistance to third-generation cephalosporins is of particular clinical significance. This genotypic screening result contributes new surveillance data on vegetable-associated bacteria in Burkina Faso — an environmental compartment not previously included in national AMR monitoring. A representative gel image from multiplex PCR is shown in Fig 3; gene prevalence among the analysed subset is illustrated in Fig 4.

**Table 2:** Distribution of β-lactamase genes among the eight resistant isolates selected for multiplex PCR analysis.

Gene	Amplicon size (bp)	No. positive / 8 analysed (%)	Interpretation
*blaTEM*	807	8/8 (100%)	Predominant β-lactamase determinant in resistant isolates; TEM-family variants can also encode ESBL activity (not confirmed phenotypically here)
*blaOXA*	619	4/8 (50.0%)	Frequent secondary β-lactamase determinant; certain OXA-family variants can also encode ESBL activity (not confirmed phenotypically here)
*blaCTX-M*	592	4/8 (50.0%)	ESBL-type gene; reduces susceptibility to 3rd-gen. cephalosporins
*Co-occurrence (all 3 genes)*	—	3/8 (37.5%)	Multi-enzyme resistance profile

**Fig 3:**
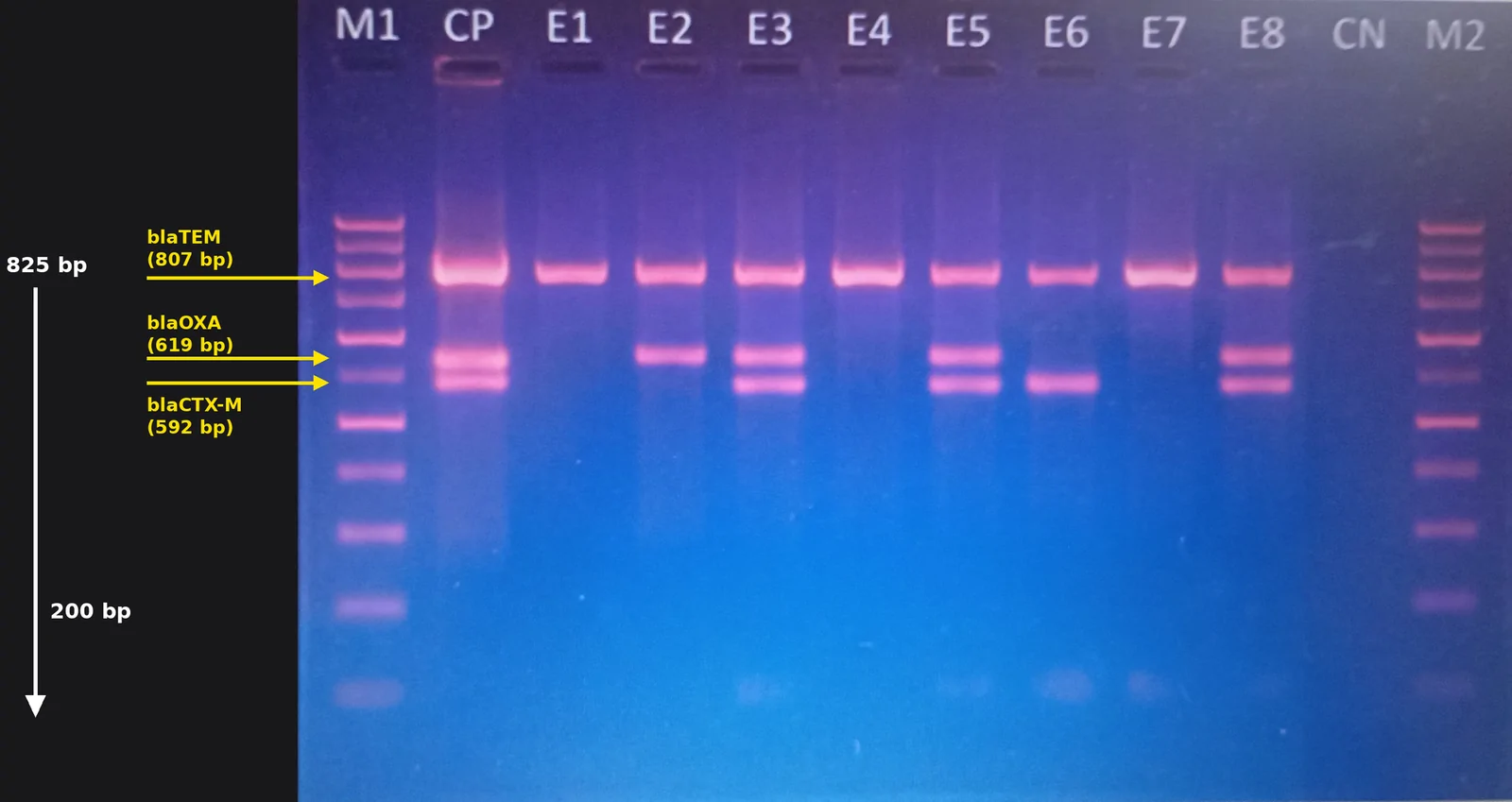
Representative multiplex PCR gel image (1.5% agarose, ethidium bromide) showing detection of *blaTEM* (807 bp), *blaOXA* (619 bp), and *blaCTX-M* (592 bp) in resistant bacterial isolates from fresh vegetable samples. Lane M1 and M2: 100 bp DNA ladder (200–825 bp range indicated); Lane CP: positive control (known ESBL-producing reference strain; all three bands present); Lane CN: negative control (sterile water; no amplification); Lanes E1–E8: test isolates showing various combinations of β-lactamase gene amplicons.

**Fig 4:**
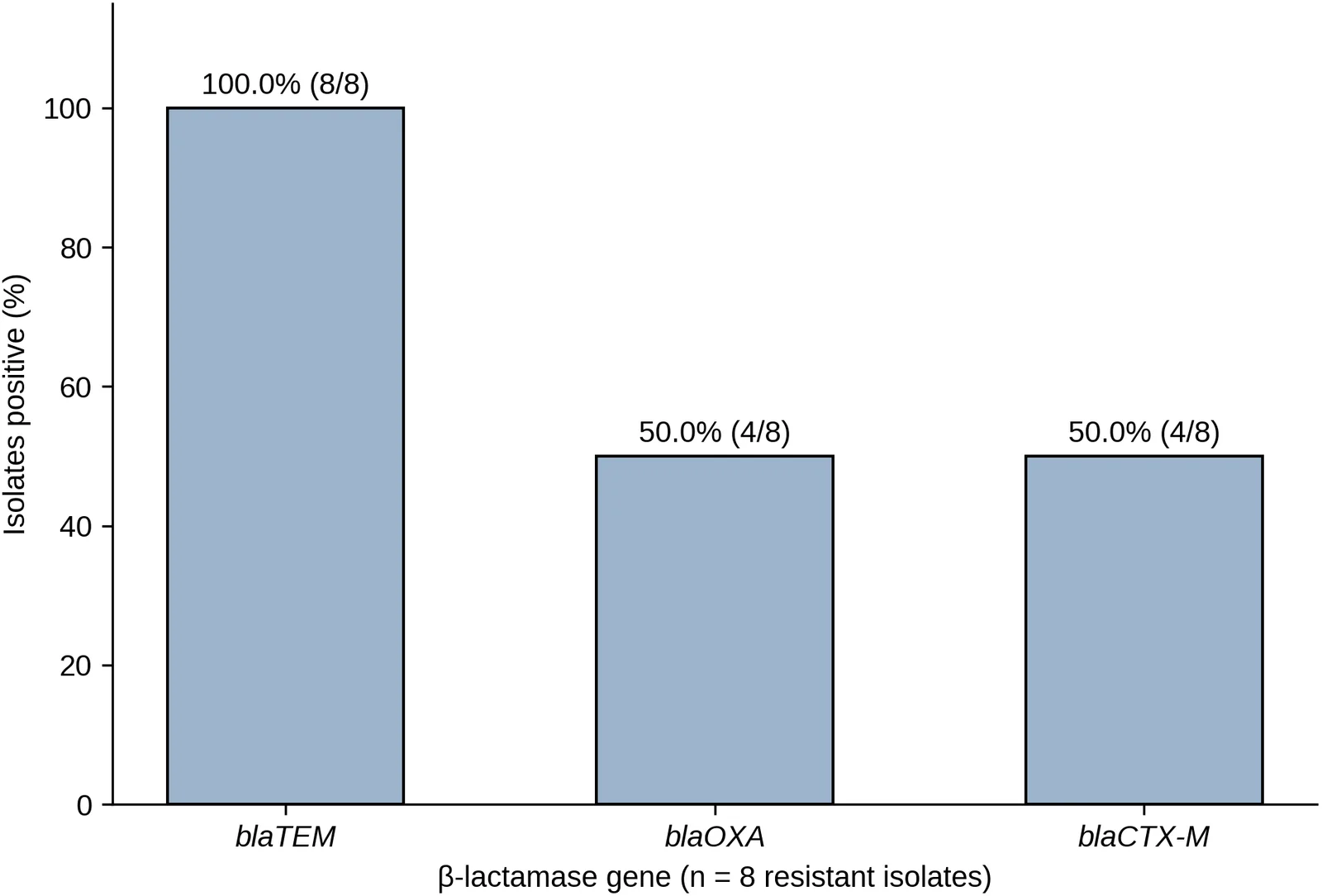
Prevalence of selected β-lactamase genes (blaTEM, blaOXA, blaCTX-M) detected by multiplex PCR among the eight-representative resistant bacterial isolates from fresh vegetable samples in Ouagadougou, Burkina Faso. Values indicate the percentage of the eight analysed isolates positive for each target gene.

### Hygiene and handling practices

The questionnaire survey revealed systemic food safety deficiencies across all sampling contexts (Table 3 and Fig 5). Ambient-temperature storage of vegetables assessed by direct observation and self-report without instrumental measurement was recorded in 98% of vending settings. No disinfection step prior to consumption or sale was applied in 85% of cases, with water rinsing used as the sole decontamination measure. Cross-contamination risk between raw vegetables and raw animal products or contaminated surfaces was identified in 80% of settings. These deficiencies were present across farms, markets, restaurants, and supermarket settings.

**Table 3:** High-risk food hygiene and handling practices identified among vendors, food-service operators, and consumers surveyed in Ouagadougou, Burkina Faso (n = 450 respondents).

High-risk practice	Frequency (%)	Food safety implication
Ambient-temperature storage (observation and self-report; no instrumental measurement)	98	Bacterial persistence and possible growth during prolonged display
No vegetable disinfection before consumption or sale	85	Failure to remove or inactivate surface-associated contaminants
Cross-contamination risk with raw animal products or surfaces	80	Transfer of enteric pathogens to ready-to-eat vegetables

**Fig 5:**
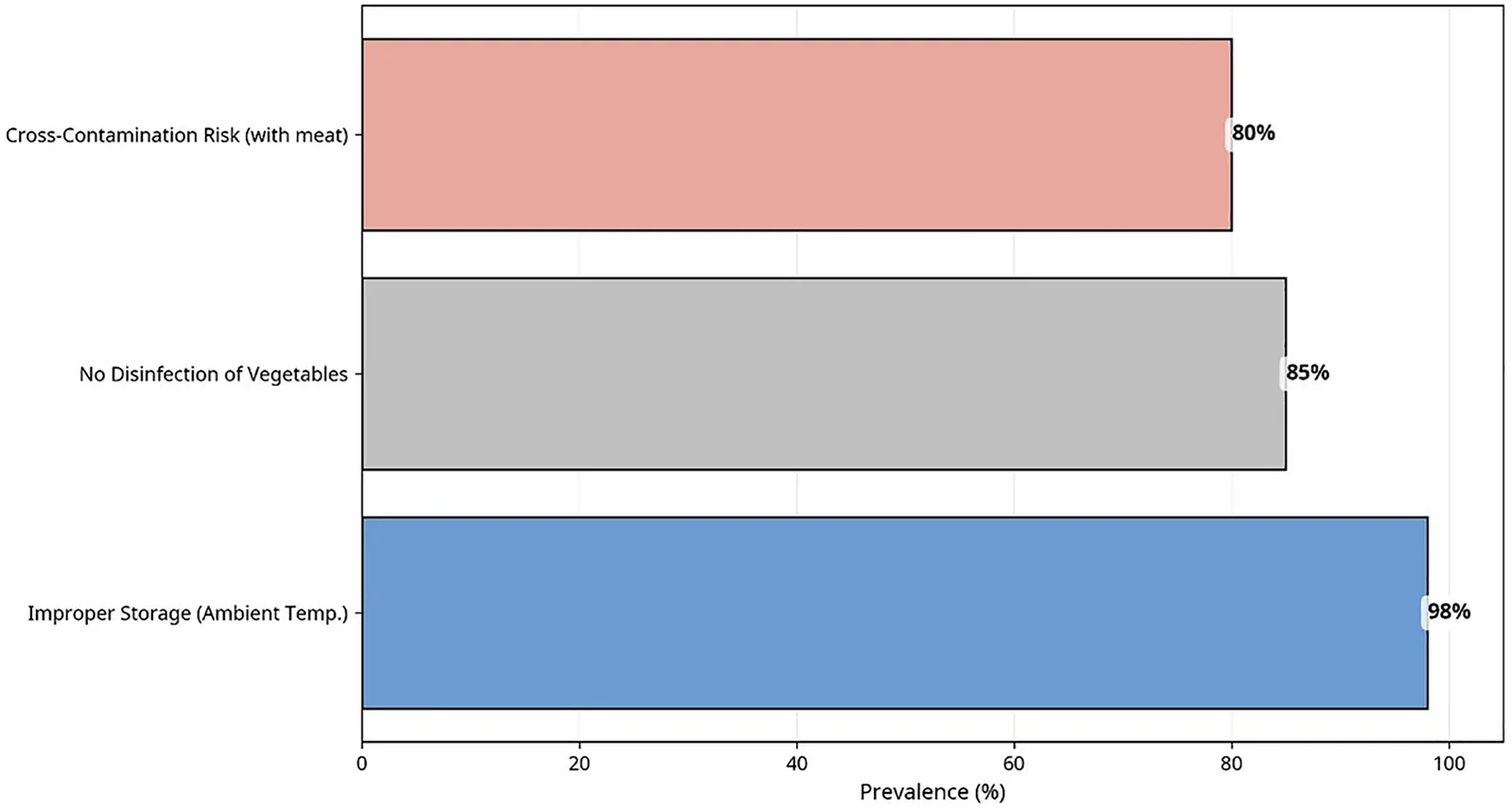
Prevalence of three key high-risk food hygiene practices among vendors, food-service operators, and consumers surveyed in Ouagadougou, Burkina Faso (n = 450 respondents). Storage temperature was assessed by direct observation and self-report; no instrumental measurements were performed.

### Screening-level risk estimate

Applying the screening-level formula Pi = Pc × Tc, with Pc = 10.0% (prevalence of *Salmonella*-positive samples) and Tc = 20.0% (estimated transmission factor). The screening-level probability of infection with antibiotic-resistant *Salmonella* per raw salad serving was estimated at Pi = 2.0% (Table 4). This corresponds approximately to one infection event per 50 servings under the simplified assumptions of the model. As specified in the Methods, this value is a screening-level indicator only and should not be interpreted as a quantitative prediction of population infection rates.

**Table 4:** Parameters used for the screening-level risk estimate.

Param.	Description	Value	Source
*Pi*	Screening-level probability of infection with resistant Salmonella per serving	2.0%	Calculated: Pc × Tc
*Pc*	Prevalence of Salmonella spp.-positive samples (of 300 total samples)	10.0%	This study
*Tc*	Transmission factor (estimated from proportion of high-risk practice settings)	20.0%	Survey-based estimate

### HACCP-based control framework

Based on the microbiological results, resistance profiles, and survey findings, a HACCP-based control framework was developed identifying five Critical Control Points (CCPs) across the fresh vegetable supply chain in Ouagadougou (Table 5 and Fig 6). CCP 1 targets agricultural production and irrigation water quality; CCP 2 addresses harvesting and transport hygiene; CCP 3 concerns market vending and storage conditions; CCP 4 covers restaurant and household vegetable washing and disinfection; and CCP 5 encompasses microbiological surveillance. This framework is proposed as an evidence-based practical tool for local food safety stakeholders and requires operational field validation before formal adoption.

**Table 5:** HACCP-based control framework for the fresh vegetable supply chain in Ouagadougou, Burkina Faso.

CCP	Main hazard	Control point	Monitoring indicator	Corrective action
**CCP 1 — Agricultural production**	Contamination via untreated irrigation water or organic fertilizer	Irrigation water quality and fertilizer management	Monthly microbial testing of water; composting process inspection	Treat irrigation water; prohibit untreated wastewater; improve manure composting
**CCP 2 — Harvesting & transport**	Cross-contamination from hands, crates, and surfaces	Handler hygiene and container sanitation	Handwashing compliance audit; container cleanliness inspection	Sanitize crates; train harvest workers; separate clean and soiled materials
**CCP 3 — Market vending**	Bacterial persistence during ambient-temperature display; cross-contamination	Storage conditions and physical separation from meat	Observation of storage conditions; separation from raw meat	Promote shaded cool storage; reduce holding time; enforce physical separation from meat
**CCP 4 — Restaurant / household**	Inadequate washing and absence of disinfection before raw consumption	Vegetable washing and disinfection protocol	Use of potable water; application of validated disinfection procedure (chlorine 50–100 ppm)	Standardize chlorine wash protocol; prevent recontamination after disinfection
**CCP 5 — Microbiological surveillance**	Absence of routine monitoring to detect contamination trends	Periodic microbiological testing and hygiene audits	Monthly microbiological testing frequency; quarterly hygiene audit compliance rate	Implement routine surveillance; communicate results to public health authorities

**Fig 6:**
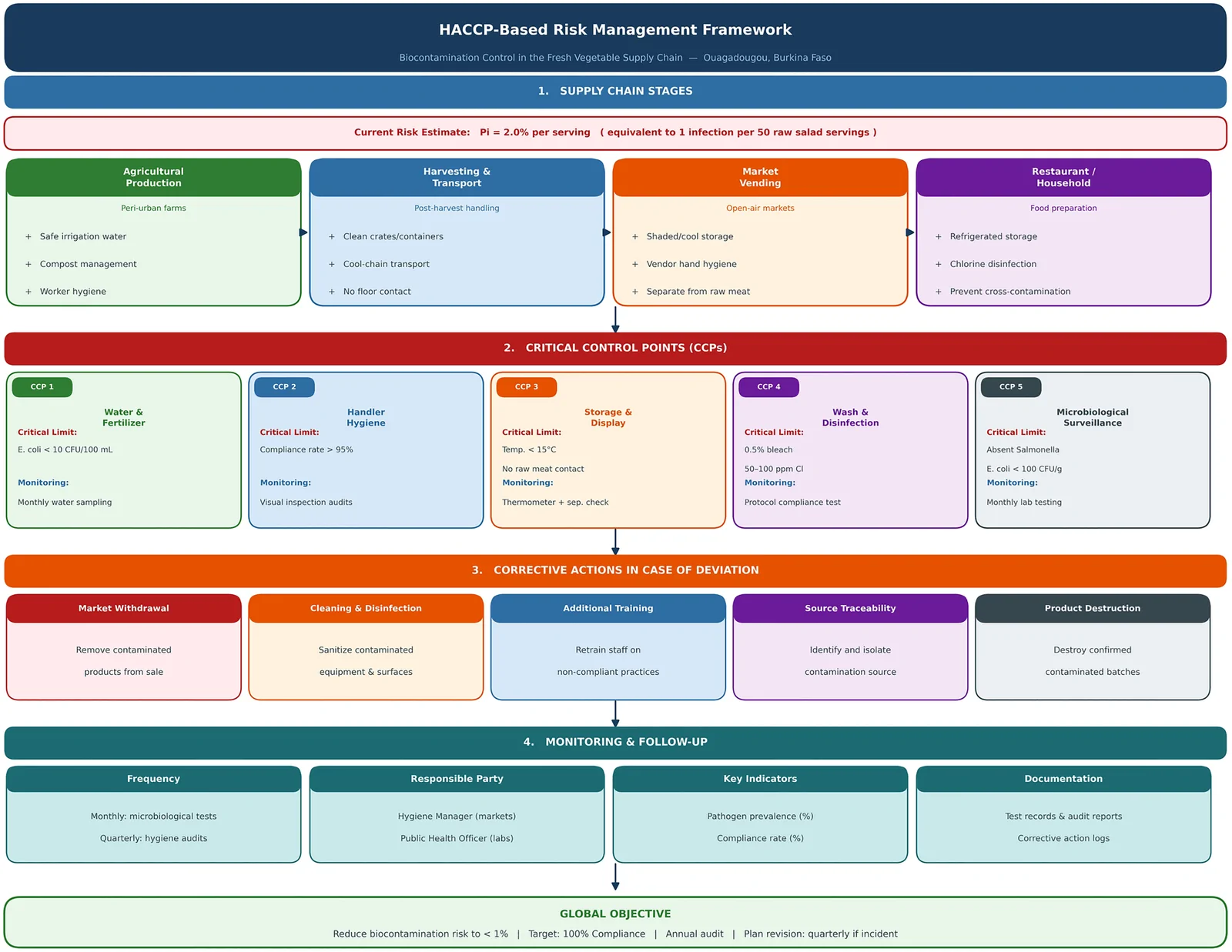
HACCP-based risk management framework for biocontamination control in the fresh vegetable supply chain in Ouagadougou, Burkina Faso. The framework identifies four main supply chain stages (Agricultural Production, Harvesting & Transport, Market Vending, Restaurant/Household Preparation), five Critical Control Points (CCPs 1–5) with critical limits and monitoring indicators, five corrective action categories, and a monitoring and follow-up structure with a global objective of reducing biocontamination risk to below 1%.

## Discussion

This study provides evidence that fresh vegetables sold and consumed in Ouagadougou carry AMR enteric bacteria with clinically relevant resistance determinants. Systemic failures in food hygiene along the supply chain create conditions favourable to bacterial persistence and consumer exposure. The sample-level prevalence of E. coli (63.0%) and Salmonella spp. (10.0%) is consistent with data from comparable West African urban settings: E. coli prevalences of 40–80% and Salmonella prevalences of 5–18% have been reported in fresh produce from Ghana, Nigeria, and Ethiopia, typically linked to contaminated irrigation water and open-air market conditions [8,26]. Detection of both pathogens across all four sampling contexts confirms that contamination is not limited to a single critical point. It is instead introduced and maintained at multiple stages of the supply chain — a pattern that requires the supply chain-wide HACCP approach proposed here. This distributed pattern of contamination reinforces a case made elsewhere in the region: fragmented, single-sector AMR surveillance is insufficient. Coordinated, multisectoral One Health surveillance across agriculture, food handling, and public health is needed to justify the resources invested and translate detection into intervention [27].

The higher resistance of Salmonella isolates than E. coli isolates for several individual agents (Table 1), together with MDR in 60% of antibiotic-resistant Salmonella isolates, carry direct clinical implications. High resistance to tetracycline (25.0%) and trimethoprim-sulfamethoxazole (21.7%) in Salmonella mirrors patterns documented in foodborne isolates from sub-Saharan Africa, reflecting sustained selective pressure from widespread use of these affordable antibiotics in human and veterinary medicine [11,13]. The detection of resistance to ciprofloxacin (4.1%) and gentamicin (5.8%) among Salmonella isolates is noteworthy, as these agents are also used as second-line treatments for enteric infections in resource-limited settings [28,29]. The contrast between Salmonella and E. coli resistance patterns may reflect differences in transmission routes and antibiotic exposure history, as has been documented in other food-chain studies in the region [26].

The molecular detection of β-lactamase genes provides the most clinically significant findings. The universal presence of *blaTEM* (100%) in resistant isolates confirms the baseline selective pressure for β-lactam resistance, consistent with *blaTEM* being the most prevalent β-lactamase determinant in Enterobacteriaceae globally [9]. The detection of *blaCTX-M* in 50.0% (4/8) of the analysed isolates is of greater clinical and public health concern. CTX-M-type ESBLs represent a globally disseminated class of resistance enzymes, now predominating in Africa, with CTX-M-15 the dominant variant in West African clinical and environmental isolates [30,31]. Their presence in vegetable-associated bacteria in Burkina Faso creates a plausible food-chain route for community-level acquisition of ESBL-producing strains, potentially compromising treatment of severe enteric infections with third-generation cephalosporins critical last-resort agents in settings where carbapenem access is limited [32]. The co-occurrence of *blaTEM*, *blaOXA*, and *blaCTX-M* in 37.5% (3/8) of analysed isolates indicates multi-enzyme resistance profiles with the broadest phenotypic coverage. Although based on a small purposively selected subset, these findings are consistent with the regional AMR literature [33] and represent, to our knowledge, a first characterization of β-lactamase genotypes in vegetable-associated bacteria in Burkina Faso.

The screening-level estimate of a 2.0% probability of infection with antibiotic-resistant Salmonella per raw salad serving provides a tangible communication tool linking the documented hygiene failures to a concrete risk signal. This estimate falls within the 0.5–5% range reported by comparable screening-level estimates for *Salmonella* in raw produce from other settings [25]. It is built directly on the survey findings: near-universal ambient-temperature storage (98%), absence of disinfection in 85% of settings, and widespread cross-contamination risk (80%) collectively create conditions for pathogen persistence from production to consumption [8,9]. While this figure is a screening-level indicator rather than a precise quantitative prediction, it underlines the relevance of the contamination documented here for public health. The HACCP-based framework proposed targeting five CCPs with specific critical limits (e.g., *E. coli* < 10 CFU/100 mL in irrigation water; chlorine 50–100 ppm for vegetable washing) and a global objective of reducing biocontamination below 1% provides actionable, evidence-grounded risk reduction measures adapted to the local informal food system [34–36].

From a One Health perspective, the food contamination, ESBL gene circulation, and inadequate hygiene infrastructure documented here converge on a single conclusion. Fresh produce needs to be integrated into national AMR surveillance frameworks linking agriculture, food safety, and human health, in Burkina Faso and across West Africa [12,13,29,37]. Several limitations should be acknowledged, structured here by domain. Molecular scope: the analysis was restricted to genotypic screening for three β-lactamase genes in eight purposively selected isolates (not the full resistant-isolate panel), without phenotypic ESBL confirmatory testing, limiting epidemiological generalization of the gene-prevalence figures. Design: the cross-sectional design precludes causal inference. We found no statistically significant dry-versus-rainy-season difference in prevalence or resistance for either species (see Results); however, the study was not powered a priori to detect smaller seasonal effects, and finer-grained (e.g., monthly) temporal patterns could not be assessed. Measurement: storage temperature was assessed by direct observation and self-report only, without instrumental measurement, which may introduce misclassification. Risk model: the screening-level risk estimate is a simplified, single-point indicator without dose-response parameters, pathogen enumeration, or uncertainty analysis, and should not be read as a validated infection probability. Future research should prioritise: longitudinal, season-stratified sampling; pathogen enumeration and dose-response-based quantitative microbial risk assessment; whole-genome sequencing and phenotypic ESBL confirmation across the complete resistant-isolate panel; and prospective field evaluation of the proposed HACCP framework’s effectiveness in operational settings.

## Conclusions

Fresh vegetables sold in Ouagadougou, Burkina Faso carry AMR enteric bacteria harboring clinically relevant β-lactamase genes including the ESBL-associated *blaCTX-M* determinant, alongside widespread high-risk food hygiene practices. A screening-level exposure-risk indicator for antibiotic-resistant *Salmonella* per raw salad serving (2.0%; not a confirmed infection probability, see Methods) underscores the public health relevance of these findings. Three key messages emerge. First, fresh vegetables are a significant and underappreciated vehicle for AMR foodborne pathogens in this West African urban context. Second, the genotypic detection of ESBL-associated genes in vegetable-associated bacteria contributes new surveillance data for Burkina Faso and signals the need for expanded genomic and phenotypic AMR surveillance. Third, an integrated One Health approach is essential to reduce consumer risk, combining routine fresh produce microbiological surveillance, food handler training, improved hygiene infrastructure, and a preliminary, field-validation-pending HACCP framework with five identified CCPs. Coordinated commitment across agricultural, food service, and public health sectors is required for sustained impact.

## Supporting information

S1 Raw images Original, uncropped and unadjusted multiplex PCR gel image underlying Fig 3. Lane order: M1 (DNA ladder), CP (positive control), E1–E8 (test isolates), CN (negative control), M2 (DNA ladder).
(PDF)
