# Supplementary material for: Antimicrobial-resistant Salmonella and Escherichia coli in fresh vegetables from Ouagadougou, Burkina Faso: Prevalence, β-lactamase genes, and food safety implications
Source: PLoS One. 2026 Nov 5;21(11):e0361890. doi: 10.1371/journal.pone.0361890 (PMC13739176; doi:10.1371/journal.pone.0361890)
Supplement: Supplementary file 1 [file pone.0361890.s001.pdf]

S1_raw_images. Original multiplex PCR gel image underlying Fig 3 (PONE-D-26-27447R1)

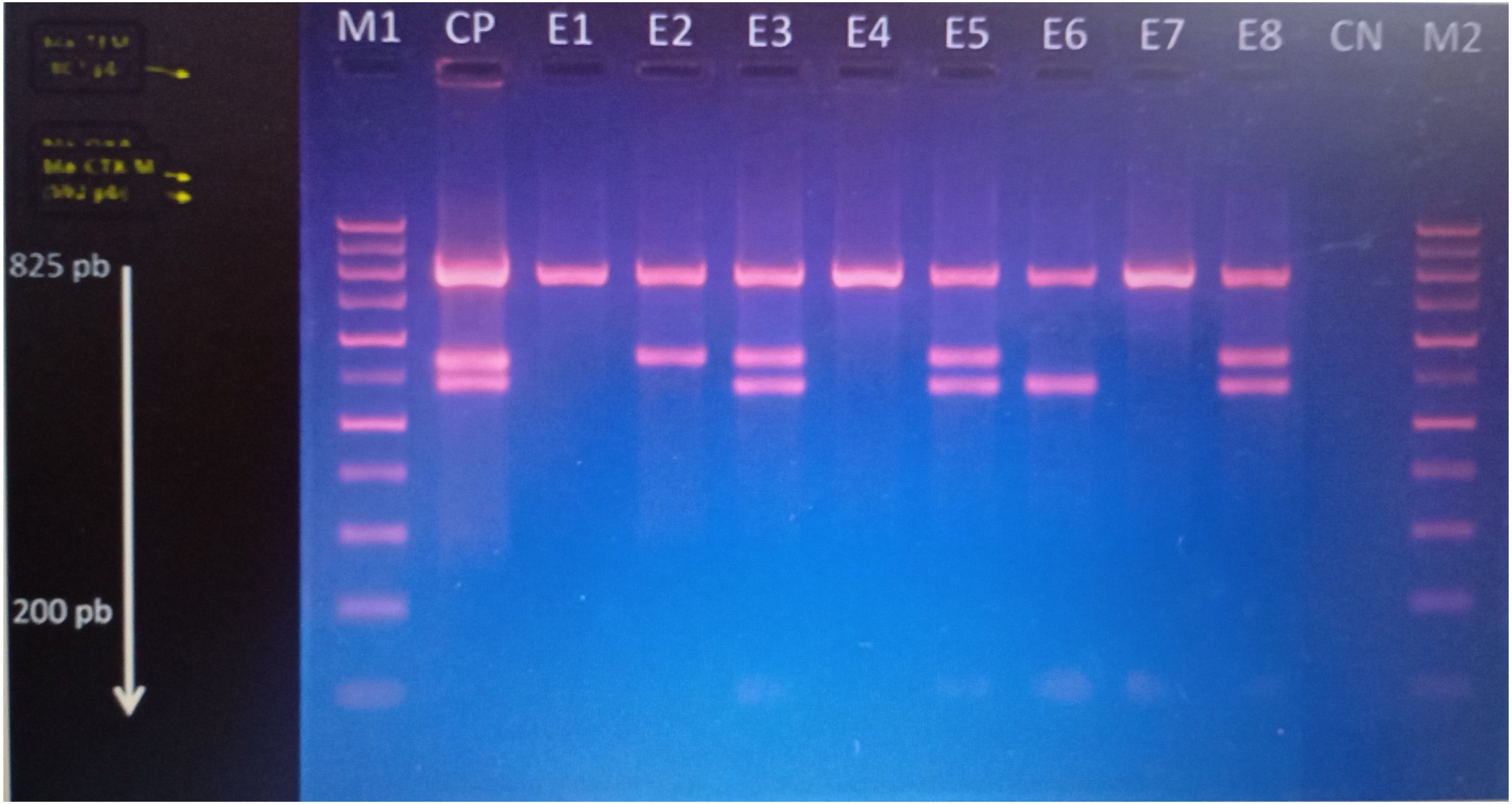

Image shown exactly as captured (no cropping, no adjustment). Lanes (left to right): M1 (DNA ladder), CP (positive control), E1-E8 (test isolates), CN (negative control), M2 (DNA ladder).

Gel: 1.5% agarose, ethidium bromide, UV visualisation. Targets: blaTEM (807 bp), blaOXA (619 bp), blaCTX-M (592 bp). All lanes on the gel are shown in Fig 3; no lane was removed (no lane marked X).

Ladder: 100 bp DNA ladder (lanes M1 and M2; 200-825 bp range indicated in Fig 3). Used in: Fig 3 (this image is the complete source of the figure).

Capture method: photograph of the gel-documentation display after UV transillumination (the original instrument file was not retained).
